# An analysis of the textural properties of activated carbons obtained from biomass via the LBET, NLDFT and QSDFT methods

**DOI:** 10.1038/s41598-024-76297-x

**Published:** 2024-11-02

**Authors:** Mirosław Kwiatkowski

**Affiliations:** Faculty of Energy and Fuels, AGH University of Krakow, al. Adama Mickiewicza 30, Krakow, 30-059 Poland

**Keywords:** Biomass, Adsorption, Desorption, Heterogeneity, Micropores, Isotherms, Materials for energy and catalysis, Structural materials, Techniques and instrumentation, Theory and computation, Physical chemistry, Surface chemistry, Chemical synthesis

## Abstract

This article presents the unique research results of the comprehensive analysis of the porous structure of activated carbons obtained from biomass waste materials from the wood industry during activation in an air atmosphere. The porous structure was analysed on the basis of nitrogen and argon adsorption isotherms via complementary multi-method analysis, i.e. the new numerical clustering-based adsorption analysis, the non-local density functional theory and the quenched solid density functional theory methods. The analytical results for the prepared activated carbons were compared with analogous results obtained for commercial activated carbon. On the basis of the conducted studies it has been determined that the new numerical clustering-based adsorption analysis method gives credible and valuable information on the textural properties of activated carbons which are in strict correlation and mutually complement with the results of the analysis with the use of the quenched solid density functional theory method. The research results obtained in this paper, it has also been shown that from waste materials of the wood industry, in a relatively cheap and cleaner production process, it is possible not only to obtain carbonaceous materials almost comparable to commercial activated carbon, but also to manage the waste in accordance with the principles of a closed-loop economy and sustainable development. The paper pays also attention to the often overlooked economic and ecological aspects, which should nevertheless be taken into account when comparing different adsorbents, rather than their textural properties alone.

## Introduction

Environment pollution is one of the main problems of modern civilization, caused by the rapid development of industrial activities and significant population growth. Therefore, many measures are being taken to stop this unfavourable trend. One of the most popular methods of removing pollutants is the adsorption method, and activated carbons are among the most popular and commonly used adsorbents both in industrial processes and in everyday life, thanks to their unique textural properties, including a large specific surface area and considerable pore volume^1–4^.

The continuous development of new technologies is constantly expanding the areas of application of porous activated carbons and intensifying the pace of research to improve their production processes^5–9^.The use of activated carbons in industrial processes entails, inter alia, the need to constantly reduce the cost of their production, in order to ensure and maintain competitiveness in relation to other technologies and materials.

Lowering production costs and increasing its efficiency is possible both by replacing raw materials with their cheaper counterparts, often with waste materials and by optimizing their production conditions^10^. High competitiveness of activated carbons in relation to other adsorbents is also ensured by the possibility of manufacturing a wide range of products, dedicated to various applications. Great attention is also paid to the environmental aspects of the production of activated carbons, including the reduction of the negative environmental impact of the technology of their production, as well as the reduction of its energy intensity^11–13^.

Activated carbons are produced from materials such as hard and brown coal, plastics, biomass, and many others^14–18^. The textural properties of carbonaceous adsorbents depend significantly on the porous structure and chemical structure of the original raw material^19^. As a result, the selection of the right material is no less important than choosing the right production technology and optimally determining the appropriate manufacturing process conditions. Therefore, the search for new raw materials that would be useful in the production of carbonaceous adsorbents has begun.

In the selection of the material for the production of activated carbons, also the availability of the raw material and the cost of its acquisition are taken into account in addition to its physical and chemical properties. Hence, biomass of agricultural, forest and food-industry origin is increasingly commonly used for the production of activated carbons, which enables the conversion of generally available, worthless and often troublesome waste into valuable products used, among others, in environmental protection^20–25^.

Activated carbons are produced by a carbonisation and a subsequent physical or chemical activation^26^. At an early stage of carbonisation process, i.e., at a temperature of around 300–350 °C, less permanent bonds in the polymer network break down, and free macromolecules are formed. At the same time, significant amounts of volatile components are discharged, and the carbon atoms are grouped into a more stable form. Connections between macromolecules also form, and the beginnings of a porous structure develop. When the carbonisation temperature exceeds 500 °C, the structure formed becomes increasingly carbonised and aromatised due to eliminating hydrogen and oxygen atoms^27–29^.

The carbonisation process is one of the most important stages in the producing activated carbons, as it determines the formation of the primary porous structure. The primary porous structure or its initial form produced during carbonisation is only developed in the initiated direction during activation, and it is usually impossible to make a major changes to this structure through a corresponding selection of activation parameters. However, the product of the carbonisation process is characterised by a poorly developed porous structure and must, therefore, be subjected to a physical or chemical activation process^27–29^.

Physical activation involves partial gasification of the char by oxidising agents, i.e., most commonly steam or carbon dioxide at 800–1000 °C, or less commonly oxygen at temperatures below 800 °C^30,31^. The reactions occurring during the activation process produce gaseous products and a gradual reaction of the carbonaceous substance, which is replaced by voids called pores, while the specific surface area of the material increases.

The greatest influence on the activation process is temperature, i.e., at low temperatures the rate of chemical reaction of the carbonaceous substance with the oxidising agent is low, as is the overall process rate. Under these conditions, a dynamic equilibrium is established between the concentration of the oxidising agent in the pores and its concentration in the space between the grains, resulting in a homogeneous product with a uniform pore size distribution. On the other hand, an increase in temperature of activation can induce uncontrolled intensive oxidation, which usually leads to an undesirable reduction in the volume of the micropores due to wall firing between adjacent pores, which in turn results in a deterioration of both the adsorption and mechanical properties of the final product^30,31^.

At very high temperatures, the oxidation rate increases to the point where virtually all of the oxidising agent reacts with the carbonaceous substance only on its outer surface, and no porous structure is formed at all^30,31^. The rate of oxidation during activation is also limited by the reactivity of the carbonaceous substance and the activity of the gaseous oxidising agent; the higher the reactivity of the carbonaceous substance and the activity of the oxidising agent, the lower the process temperature at which homogeneous development of the porous structure is observed. The formation of a porous structure also depends on the nature of the activating agent, i.e., the use of carbon dioxide as an activating agent result in a better development of micro- and ultramicroporous structures, while the use of steam results in the development of a structures with a wider pore size distribution and a high proportion of mesoporous structures^30,31^.

The advantages of physical activation include relatively low costs of producing activated carbons and the possibility of preserving the shape and texture of the raw material, which enables the production of, among others, activated nonwovens and activated carbon fabrics^30,31^. However, the physical activation process produces carbonaceous adsorbents whose specific surface area usually does not exceed 2000 m^2^/g. In addition, there is a significant weight loss of up to 70%, and two separate high-temperature carbonisation and activation processes generate high energy costs. Consequently, new methods of producing activated carbons with lower production costs are being sought, including, among other things, lower energy consumption for their production^32^.

## Materials and methods

Analyzed in this paper the activated carbons were prepared by carbonization raw biomass materials under Ar atmosphere in temperature 800 °C, physical activation in air in temperature 300 °C and finally carbonized under Ar at 800 °C. The starting materials were: Pine, Oak, and Beech woods waste. The raw materials were placed in the quartz tube, heated 10 °C/min up to 800 °C, then cooled down to room temperature where the atmosphere was changed to air. Then samples were heated 10 °C/min up to 300 °C and holded for 3 h, and next finally carbonized under argon atmosphere at 800 °C by heating 10 °C/min up to 800 °C.

The obtained samples have been marked with names that come from the name of the original raw material (i.e. the activated carbon obtained from Pinewood has been named PAC, and analogically, activated carbons obtained from Oakwood OAC, and activated carbons obtained from Beechwood BAC). The textural properties of activated carbons obtained from biomass materials were compared with those of commercial activated carbon Norit R 0.8.

The use of the activated carbons requires their characterization, which comprises, among others description of porosity, surface texture and energetic heterogeneity. Physical adsorption of different gases became a most popular method for the characterization of textural properties of the activated carbons. The common use of nitrogen for the determination of adsorption isotherms has been criticised, which is related to the fact that the value of the settlement surface area of the nitrogen molecule of 0.162 nm^2^, assumes that the nitrogen molecule adsorbs flat on the adsorbent surface. In fact, the functional groups interacting with the nitrogen quadrupole moment lead to a change in the orientation of the adsorbed nitrogen molecule. Consequently, the sitting surface area of the nitrogen molecule of the cross-section may be much smaller than the commonly assumed value, resulting in a significant error in the estimation of the specific surface area. Unlike nitrogen, argon does not exhibit specific interactions due to its surface functional groups; being an atom, argon will always lie on the surface in the same way, regardless of the polarity of the material, thus eliminating the uncertainty of the effective cross-sectional area. Therefore, in order to assess the reliability and validity of the analytical results obtained using nitrogen adsorption isotherms argon adsorption isotherms were also determined as part of the ongoing research and a textural properties analysis was also carried out based on these.

Numerous methods of analysing the textural properties of adsorbents on the basis of adsorption isotherms of vapours and gases have been developed. The most popular methods are the BET^33^, and the DR^34^ methods, but the obtained results of textural properties description are not good enough. Therefore new methods of textural properties description are sought.

Attempts at developing universal and reliable method of textural properties description and the processes which occur on the heterogeneous surfaces have resulted in designing the new numerical clustering-based adsorption analysis methods^35–38^. Among other applications, the LBET method is dedicated to the analysis of the textural properties of heterogeneous carbonaceous materials such as activated carbons and others^35–38^. This method makes it possible to obtain a great deal of information about the analysed textural properties, including e.g. information about the volume of the first adsorbed layer, the degree of heterogeneity of the analysed surface, the size and shape of the clusters of adsorbate molecules forming in the pores, the adsorption energy distribution on the surface of the tested material, as well as the reliability of the results obtained^35–38^.

Until recently, one of the most common methods for characterising textural properties has been the NLDFT method, which is based on a model of independent gap-shaped pores with ideal graphitic walls and therefore neglects the influence of, among other things, heterogeneities and deviations from such an ideal structure in real porous materials^39^. However, the NLDFT model has a significant drawback: for carbonaceous materials with energetically and geometrically heterogeneous surfaces, artificial gaps may appear in the determined pore size distribution (PSD)^40,41^.

Efforts have therefore been made to develop a new method based on density functional theory. Ravikovitch and Neimark proposed a new model called QSDFT, which is suitable for modelling adsorption on heterogeneous carbonaceous adsorbents with corrugated amorphous walls^42^. The QSDFT method represents a significant advance in the characterisation of the textural properties of carbonaceous adsorbents based on their adsorption isotherms, particularly in the low pressure range. This is related to the fact that the reliability of the pore size distributions has increased significantly: the sharp minima in the pore size distribution curve present with the NLDFT method at ~ 1 and 2 nm no longer appear in the pore size distributions determined from the QSDFT method.

In order to obtain a wide range of complementary information on the textural properties of the obtained activated carbons, and to compare it to that of the commercial Norit R 0.8 activated carbon, an extensive analysis based on nitrogen and argon adsorption isotherms was carried out using the BET and the LBET methods, as well as based on only nitrogen isotherms was carried out using the QSDFT and NLDFT methods.

## Discussion of the results

On the basis of the obtained results analyses performed using the BET method gathered in Table 1, it has been determined that the Norit R 0.8 activated carbon has the largest specific surface area, and the activated carbon obtained from Pinewood has the smallest one. Interestingly, the value of the specific surface area of the carbonaceous material obtained from Oakwood is similar to the value obtained for the Norit R 0.8 commercial activated carbon. It should be noted, however, that the specific surface area for Norit R 0.8 activated carbon is significantly lower than that declared by the manufacturer, i.e. 1043 m^2^/g.

**Table 1:** Results of analyses of a textural properties of activated carbons using the BET and LBET methods based on nitrogen adsorption isotherms; where: *S*_BET_ is the specific surface area, Model LBET No. is the number of the best fitted LBET class model, *h* is the surface heterogeneity parameter, *V*_*hA*_ is the volume of the first adsorbed layer, *α* is the geometrical parameter of the porous structure determining the height of the adsorbate molecule clusters, *β* is the geometrical parameter of the porous structure determining the width of the adsorbate molecule clusters, *Q*_*A*_/*RT* is the dimensionless energy parameter for the first adsorbed layer, *B*_*C*_ is the dimensionless energy parameter for the higher adsorbed layers.

Material	*S*_BET_ [m^2^/g]	Model LBET No.	*h*	*V*_*hA*_ [cm^3^/g]	*α*	*β*	*Q*_*A*_/*RT*	*B*_*C*_
PAC	498.26	19	3	0.230	0.50	1.00	13.02	7.67
OAC	723.67	19	3	0.342	0.81	1.03	13.66	6.90
BAC	617.44	6	3	0.298	0.45	1.00	11.26	7.65
Norit R 0.8	798.45	4	3	0.363	0.70	1.83	12.97	7.05

**Fig. 1:**
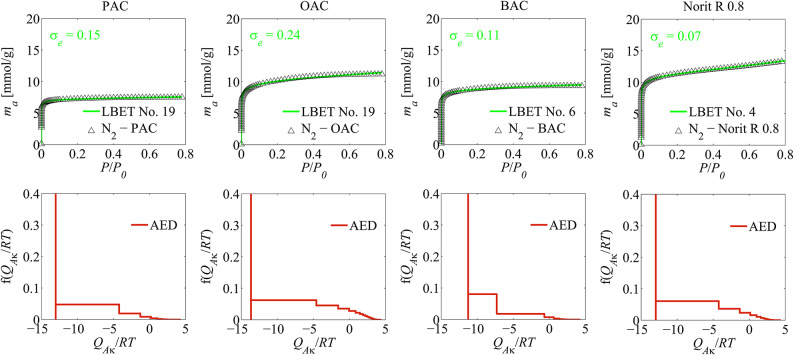
The results of the analysis of a textural properties of activated carbons, based on nitrogen adsorption isotherms, using the LBET method; where: AED is the adsorption energy distribution on the first adsorbed layer.

**Fig. 2:**
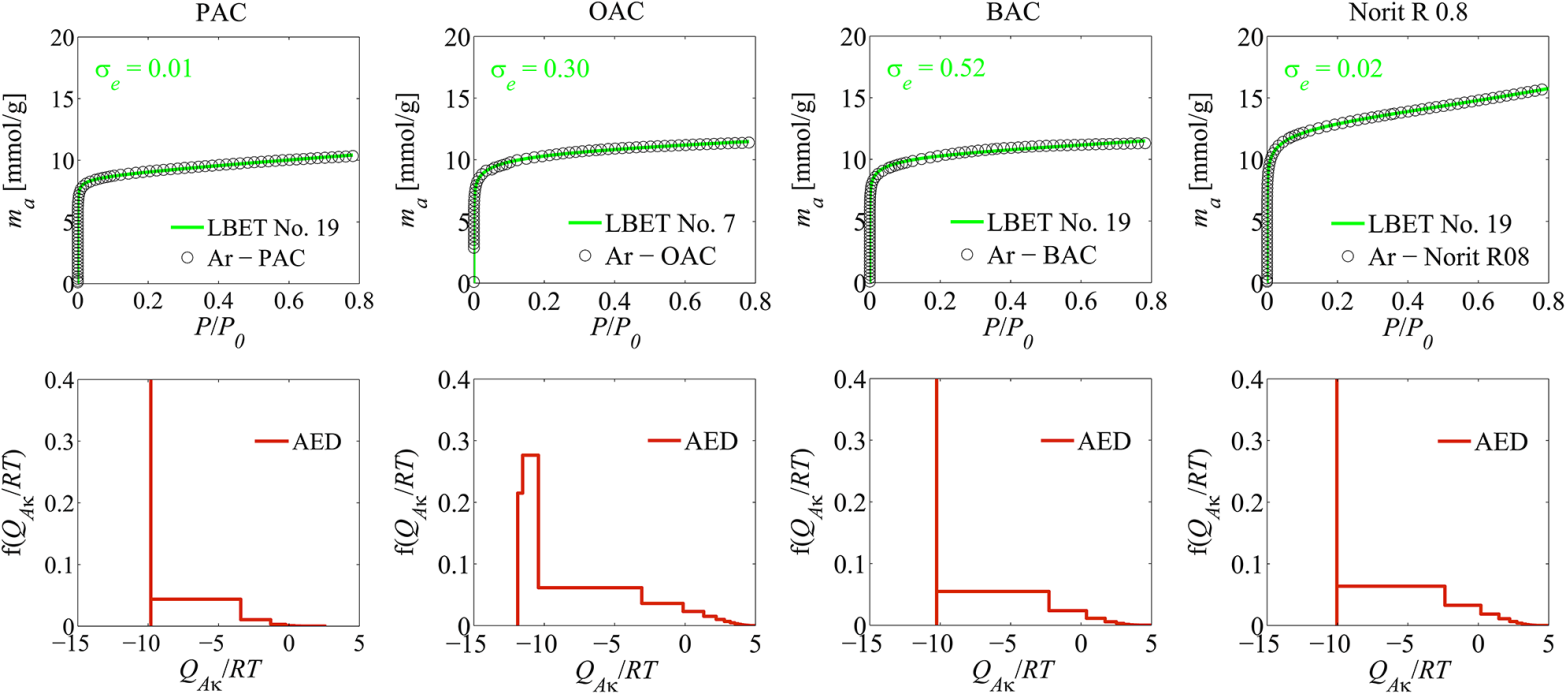
The results of the analysis of a textural properties of activated carbons, based on argon adsorption isotherms, using the LBET method; where: AED is the adsorption energy distribution on the adsorbed first layer.

On the basis of the results of the analyses a textural properties based on nitrogen and argon adsorption isotherms obtained via the LBET method presented in Tables 1 and 2 and additionally in Figs. 1 and 2, it can be stated that the analysed carbonaceous materials have identical degree of surface heterogeneity, which is proved by the values of the *h* parameter, and the values of energetic parameters, i.e. of adsorption energy for the first adsorbed layer *Q*_*A*_/*RT* and adsorption energy for upper layers *B*_*C*_ are very similar.

**Table 2:** Results of analyses of a textural properties of activated carbons using the BET and LBET methods based on argon adsorption isotherms.

Material	*S*_BET_ [m^2^/g]	Model LBET No.	*h*	*V*_*hA*_ [cm^3^/g]	*α*	*β*	*Q*_*A*_/*RT*	*B*_*C*_
PAC	557.43	19	3	0.250	0.30	1.08	−9.81	1.00
OAC	632.17	7	5	0.323	0.68	1.84	−11.88	15.87
BAC	655.13	19	3	0.313	0.52	3.07	−10.25	15.50
Norit R 0.8	812.74	19	3	0.396	0.61	3.02	−10.04	10.36

The conducted studies have shown significant differences only in values of geometric textural parameters obtained for the particular samples of activated carbons. Namely, for the Norit R 0.8 it has been indicated that high and branching adsorbate molecules clusters form in the pores of this material - it is indicated by the values of geometric parameters *α* = 0.7 and *β* = 1.83. Limitations in growth of particular adsorbate molecules clusters result from a competitive expansion of the neighbouring clusters, which is indicated by the number of the best fitted LBET class model.

The analysis of the adsorption energy distribution on the first adsorbed layer (AED) diagrams determined on the basis of adsorption isotherms of both nitrogen as well as argon, shows that there is a dominant fraction of adsorption sites where one adsorbate molecules adsorbs, and the remaining fraction of sites of a wide range of adsorption energy on the first adsorbed layer.

The analysis of the results obtained on the basis of nitrogen adsorption isotherm on the PAC activated carbon obtained from Pinewood also indicates that the examined material is microporous as well, and middle-sized, non-branching adsorbate particle clusters form in its pores - it is indicated by the parameter values *α* = 0.5 and *β* = 1.00. What is interesting however, is the significantly lower value of volume of the first adsorption layer *V*_*hA*_ = 0.230 cm^3^/g, comparing to the previously analysed Norit R 0.8 activated carbon.

On the basis of the results of the calculations with the use of the LBET method for the OAC activated carbon obtained from Oakwood it can also be stated that high, stack-like adsorbate molecules clusters form in its pores (*α* = 0.81 and *β* = 1.03), thus it can be concluded conclude that narrow, non-branching micropores are present in this material. It should be noted that the value of the volume for the first adsorbed layer obtained for the OAC sample is similar to the value obtained for the Norit R 0.8 sample. The type of the best fitted model indicates the presence of geometric growth limitations of clusters, related to the presence of narrow micropores.

The analyses of the diagram of adsorption energy distribution on the first adsorbed layer indicates, analogically to the previously discussed cases, a presence of a dominant fraction of adsorption sites of a narrow energy range of primary adsorption sites and of a remaining fraction of sites of a wide energy range, whilst as we can observe, the widest range of energy adsorption on the first adsorbed layer has been obtained in the case of the analysis of the argon adsorption isotherm. This shows us that single nitrogen molecules or argon adsorbate molecules clusters adsorb in the narrowest micropores of this material.

The last activated carbon discussed as part of the presentation was the material marked as BAC, obtained from Beechwood. The conducted studies have shown that middle-sized, stack-like, non-branching adsorbate molecules clusters form in the micropores of the BAC activated carbon, what is indicated by the values of geometric parameters *α* = 0.45 and *β* = 1.00. Type 1 which is the best-fitted LBET class model, shows growth limitations of clusters induced by competitive adsorption of the neighbouring clusters. The analysis of the diagram of adsorption energy distribution on the first adsorbed layer shows that there is a very narrow fraction of adsorption sites of high adsorption energy, and a wide range of adsorption sites of various adsorption energies, in the analysed BAC sample.

As mentioned above, in order to verify the reliability and validity of the results obtained from the nitrogen adsorption isotherms, an analogous porous structure analysis was also performed using the BET and LBET methods on the basis of the determined argon adsorption isotherms.

A comparative analysis of the results obtained on the basis of nitrogen and argon adsorption isotherms showed that the results obtained on the basis of these different gas isotherms are very close to each other, which confirms the high reliability of the results obtained on the basis of nitrogen adsorption isotherms. It should be noted, however, that the argon adsorption isotherms showed larger *S*_*BET*_, *V*_*hA*_, *α* and *β*, indicating that argon molecules adsorbed at adsorption sites inaccessible to nitrogen molecules. Analysis of diagrams of the adsorption energy distribution on the first adsorbed layer of the tested activated carbons also showed a high similarity of the determined distributions based on nitrogen and argon adsorption isotherms, which also confirms the high reliability of the results obtained from nitrogen adsorption isotherms.

**Fig. 3:**
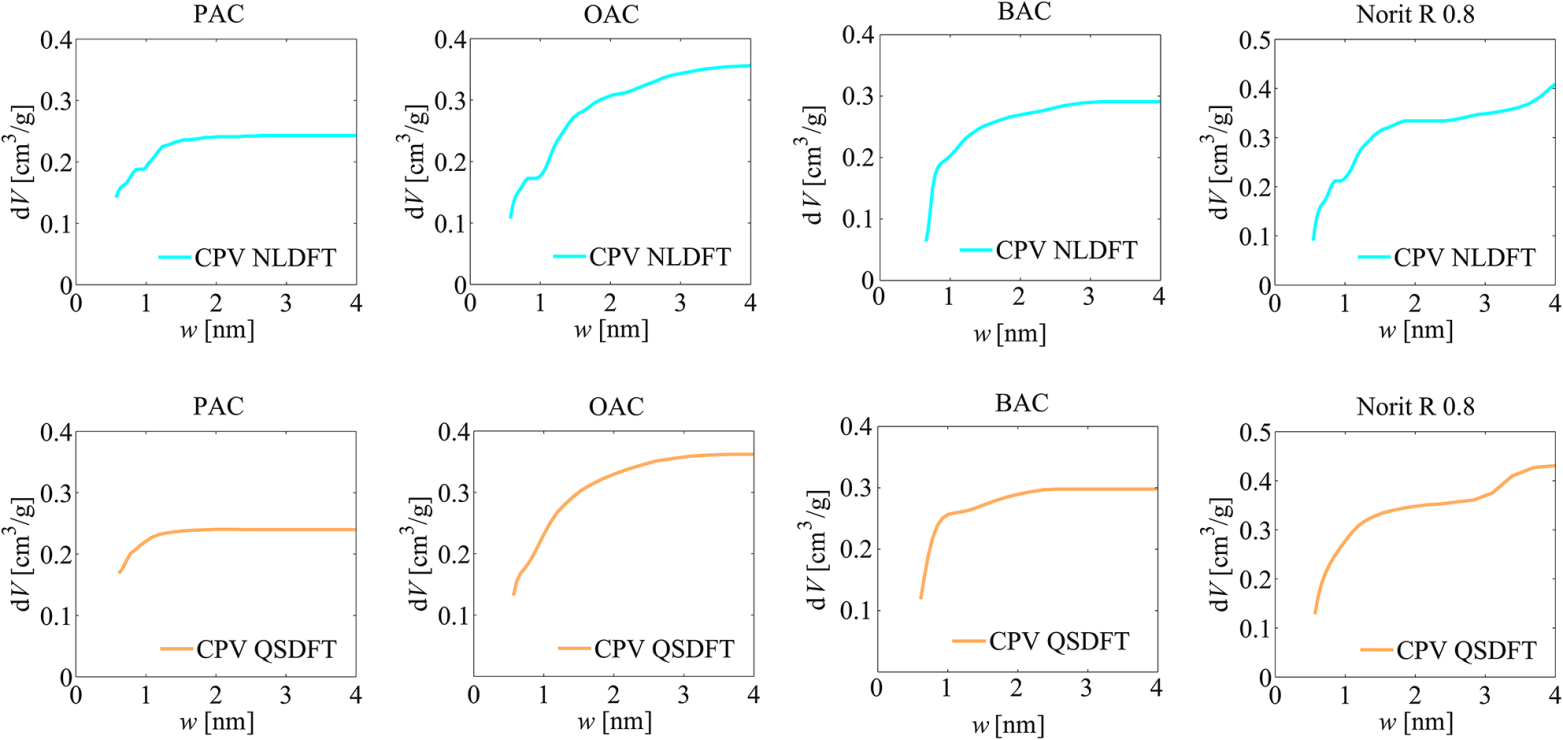
The cumulative pore volume plots obtained for the analysed activated carbons via the NLDFT and QSDFT methods.

**Fig. 4:**
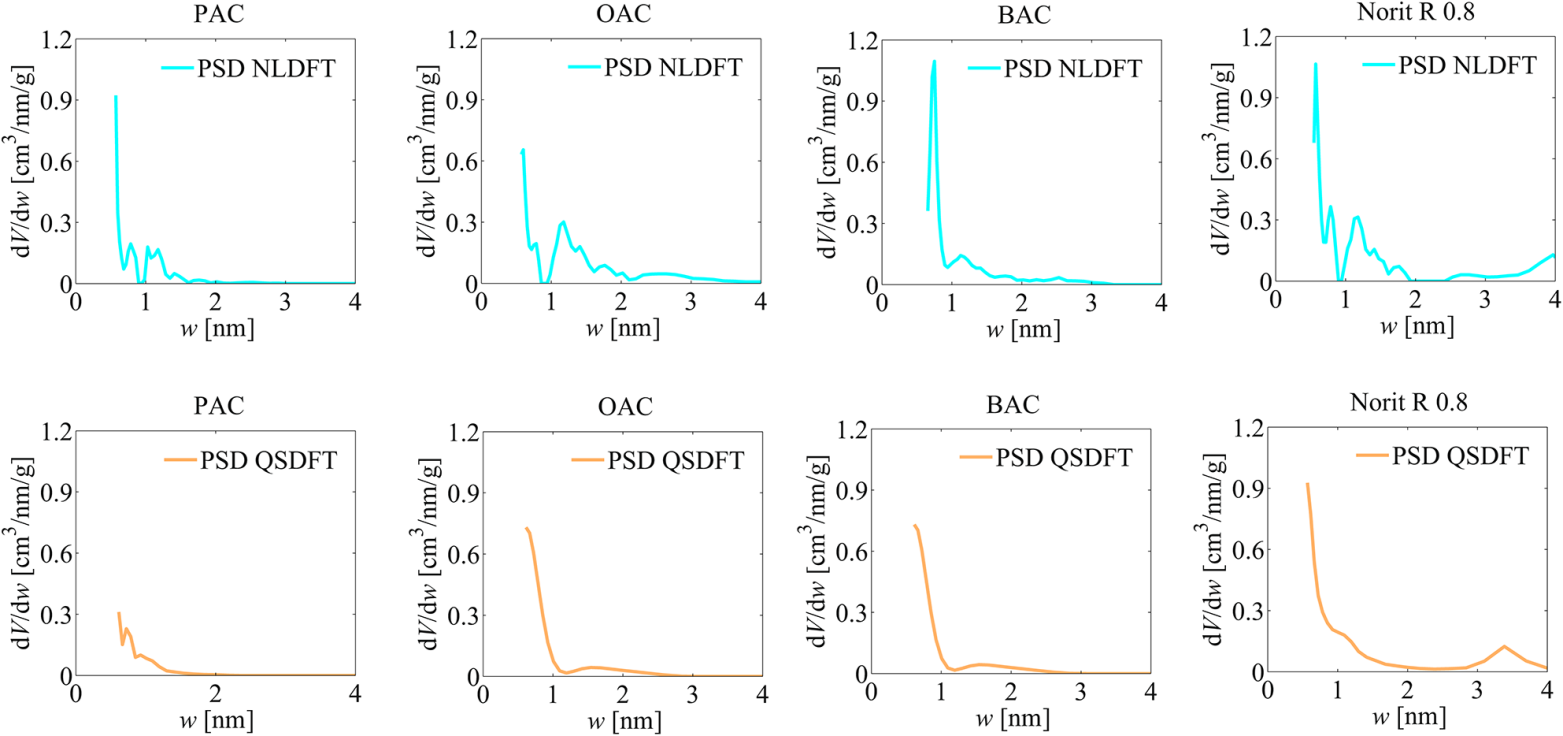
The pore size distributions plots obtained for the analysed activated carbons via the NLDFT and QSDFT methods.

Another method used to analyse the textural properties of the activated carbons was that derived from density functional theory, i.e. non localized density functional theory (NLDFT) and quenched solid density functional theory (QSDFT) methods, and the results of the analyses, i.e. pore size distribution (PSD) and cumulative pore volume (CPV) plots are shown in Figs. 3 and 4.

From the PSD and CPV distributions, it can be seen that the activated carbons obtained are characterised by a predominant proportion of micropores in the total porosity, without a significant proportion of mesopores, as observed for Norit R 0.8 activated carbon. In the PSD diagrams obtained with the NLDFT method, an artefact in the form of a break in the pore size range of approximately 0.8 to 1.0 nm is noticeable, which is due to the heterogeneity of the surface area of the materials tested, which the NLDFT pore model does not take into account.

The values of the micropores specific surface area *S*_NLDFT_ and the volume of micropores *V*_NLDFT_ obtained via NLDFT method, and the micropores specific surface area S_QSDFT_ and the volume of micropores *V*_QSDFT_ obtained via QSDFT are summarised in Table 3. Comparison of the values of the specific surface area and pore volume parameters determined using the NLDFT and QSDFT methods indicates their lower values obtained via the NLDFT method (see Table 3).

**Table 3:** The results of the analysis of a porous structure of activated carbons, based on nitrogen adsorption isotherms via the NLDFT and QSDFT method; *S*_NLDFT_ is the micropores specific surface area and *V*_NLDFT_ is the volume of micropores obtained via NLDFT method, and *S*_QSDFT_ is the micropores specific surface area and *V*_QSDFT_ is the volume of micropores obtained via QSDFT method.

Material	*S*_NLDFT_ (m^2^/g)	*V*_NLDFT_ (cm^3^/g)	*S*_QSDFT_ (m^2^/g)	*V*_QSDFT_ (cm^3^/g)
PAC	797	0.261	838	0.257
OAC	877	0.358	958	0.362
BAC	716	0.291	856	0.298
Norit R 0.8	1002	0.499	1084	0.501

## Conclusions

This article presents unique results of the studies on the textural properties of activated carbons obtained from biomass materials, with the BET, LBET, NLDFT and QSDFT methods. On the basis of the conducted research, it can be stated that the results obtained with the use of the mentioned methods are mutually complementary. Consequently, the simultaneous use of the aforementioned methods for the analysis of the porous structure guarantees that complete information on the textural properties of materials is obtained. The Norit R 0.8 activated carbon shows the greatest, and the activated carbon obtained from Pinewood - the smallest volume of micropores. The Norit R 0.8 activated carbon is also characterized by the greatest volume of mesopores. The results of the calculations performed with the use of the BET equation indicate that the Norit R 0.8 has the greatest specific surface, and the materials obtained from Pinewood – the smallest, which confirms the results obtained with the use of the QSDFT method. A high value of the specific surface obtained for the activated carbon which had been obtained from Pinewood, is close to the value obtained for the Norit R 0.8 activated carbon.

The results of analyses, obtained with the use of the LBET method, for the Norit R 0.8 activated carbon indicate that this material has the highest value of volume for the first adsorption layer which is correlated to the micropores volume obtained via the QSDFT method, and the specific surface estimated with the use of the BET method. In turn, the PAC activated carbon which showed the lowest value of micropores volume and specific surface, also had the lowest value of the first adsorption layer, estimated with the use of the LBET method.

All the methods used in the studies, including the LBET method, have indicated a significant grade of development of the microporous structure of the OAC activated carbon obtained from Oakwood, comparable to the Norit R 0.8 activated carbon. The studies with the use of the QSDFT method, have also indicated a significant similarity of the examined activated carbons in terms of small micropores, and significant differences in terms of larger micropores and mesopores.

In conclusion, although the present study has shown that the obtained activated carbons are characterised by inferior textural properties compared to the commercial activated carbon Norit R 0.8, it should be emphasised that when comparing different adsorbents comprehensively in the context of practical applications, not only their textural properties should be taken into account, but also the cost of obtaining them. In addition, consideration should be given to whether the adsorbent technology in question meets ecological requirements and fits in with the principles of sustainable development. If these aspects are considered comprehensively, it may be that materials with poorer adsorption properties, but significantly cheaper and obtained using less environmentally damaging technologies, as well as those obtained from renewable waste materials, are better for a given adsorption technique. This is due to the fact that modern adsorption techniques must compete with other solutions, and often the key aspect determining the use of a given technology is both the cost of the adsorbents used in them. At the same time ecological aspects are also increasingly important. It should be emphasised that the activated carbons in this research were obtained not only from commonly available renewable waste materials, but also by means of a relatively simple method, which is both economic and ecological, as well as, thanks to its simplicity, may be implemented anywhere in the world where a suitable raw material is commonly available.

It is worth noting that the obtained activated carbons could of course be subjected to modifications and improvements in their textual properties, but this would be pointless in the context of the main idea that guided the production of these materials, i.e. obtaining relatively good adsorbents at the lowest possible cost using cheap and simple technology, which was successfully achieved as shown by the results of the analyses presented in this paper.

## Data Availability

All data generated or analysed during this study are included in this published article.

## References

[1] Cazetta, A. L. et al. Magnetic activated carbon derived from biomass waste by concurrent synthesis: efficient adsorbent for toxic dyes. *ACS Sustain. Chem. Eng.* **4**, 1058–1068. 10.1021/acssuschemeng.5b01141 (2016).

[2] Zhou, Q. et al. Environmental perspectives of textile waste, environmental pollution and recycling. *Environ. Technol. Rev.* **11**, 62–71. 10.1080/21622515.2021.2017000 (2022).

[3] Chimanlal, I., Lesaoana, M. & Richards, H. Chemical modification of Macadamia-derived activated carbon for remediation of selected heavy metals from wastewater. *Mineral. Eng.* **184**, 107663. 10.1016/j.mineng.2022.107663 (2022).

[4] Gopinath, K. P. et al. Environmental applications of carbon-based materials: a review. *Environ. Chem. Lett.* **19**, 557–582. 10.1007/s10311-020-01084-9 (2021).

[5] Niu, H. et al. Activation of biochars by waste phosphoric acids: an integrated disposal route of waste acids and solid waste. *ACS Sustain. Chem. Eng.* **9**, 16403–16414. 10.1021/acssuschemeng.1c06326 (2021).

[6] Correa, C. R. et al. Influence of the carbonization process on activated carbon properties from lignin and lignin-rich biomasses. *ACS Sustain. Chem. Eng.* **5**, 8222–8233. 10.1021/acssuschemeng.7b01895 (2017).

[7] Blankenship, L. S. & Mokaya, R. Modulating the porosity of carbons for improved adsorption of hydrogen, carbon dioxide, and methane: a review. *Mater. Adv.* **3**, 1905–1930. 10.1039/D1MA00911G (2022).

[8] Villota, E. M. et al. Optimizing microwave-assisted pyrolysis of phosphoric acid-activated biomass: impact of concentration on heating rate and carbonization time. *ACS Sustain. Chem. Eng.* **6**, 1318–1326. 10.1021/acssuschemeng.7b03669 (2018).

[9] Kuptajit, P., Sano, N., Nakagawa, K. & Suzuki, T. A study on pore formation of high surface area activated carbon prepared by microwave-induced plasma with KOH (MiWP-KOH) activation: effect of temperature-elevation rate. *Chem. Eng. Process. Process. Intens*. **167**, 108511. 10.1016/j.cep.2021.108511 (2021).

[10] El-Nahas, S., Salman, H. M. & Saber, A. M. Production of low-price carbon for removal of aluminium ions in potable water. *J. Environ. Eng. Sci.* **16**, 145–164. 10.1680/jenes.20.00055 (2021).

[11] Varma, R. S. Biomass-derived renewable carbonaceous materials for sustainable chemical and environmental applications. *ACS Sustain. Chem. Eng.* **7**, 6458–6470. 10.1021/acssuschemeng.8b06550 (2019).

[12] Adlak, K., Chandra, R., Vijay, V. K. & Pant, K. K. Suitability analysis of sustainable nanoporous adsorbents for higher biomethane adsorption and storage applications. *Int. J. Energy Res.* **46**, 14779–14793. 10.1002/er.8182 (2022).

[13] Glogic, E. et al. Life cycle assessment of supercapacitor electrodes based on activated carbon from coconut shells. *ACS Sustain. Chem. Eng.* **10**, 15025–15034. 10.1021/acssuschemeng.2c03239 (2022).

[14] Machado, N. C. F. et al. Waste-polystyrene foams-derived magnetic carbon material for adsorption and redox supercapacitor applications. *J. Clean. Prod.* **313**, 127903. 10.1016/j.jclepro.2021.127903 (2021).

[15] Shao, J., Ma, C., Zhao, J., Wang, L. & Hu, X. Effective nitrogen and sulfur co-doped porous carbonaceous CO_2_ adsorbents derived from amino acid. *Colloids Surf. A* **632**, 127750. 10.1016/j.colsurfa.2021.127750 (2022).

[16] Lu, T. et al. Nitrogen and sulfur co-doped porous carbons from polyacrylonitrile fibers for CO_2_ adsorption. *J. Taiwan. Inst. Chem. Eng.* **128**, 148–155. 10.1016/j.jtice.2021.08.043 (2021).

[17] Cao, K. L. A., Rahmatika, A. M., Kitamoto, Y., Nguyen, M. T. T. & Ogi, T. Controllable synthesis of spherical carbon particles transition from dense to hollow structure derived from Kraft lignin. *J. Colloid Interface Sci.* **589**, 252–263. 10.1016/j.jcis.2020.12.077 (2021). 33460856 10.1016/j.jcis.2020.12.077

[18] Kayiwa, R., Kasedde, H., Lubwama, M. & Kirabira, J. B. Mesoporous activated carbon yielded from pre-leached cassava peels. *Bioresour. Bioprocess.* **8**, 53. 10.1186/s40643-021-00407-0 (2021). 38650239 10.1186/s40643-021-00407-0 PMC10991969

[19] Wang, X. et al. Layer-stacking activated carbon derived from sunflower stalk as electrode materials for high-performance supercapacitors. *ACS Sustain. Chem. Eng.* **6**, 11397–11407. 10.1021/acssuschemeng.8b01334 (2018).

[20] Mokrzycki, J., Magdziarz, A. & Rutkowski, P. The influence of the Miscanthus giganteus pyrolysis temperature on the application of obtained biochars as solid biofuels and precursors of high surface area activated carbons. *Biomass Bioenerg.* **164**, 106550. 10.1016/j.biombioe.2022.106550 (2022).

[21] Wang, L. et al. Sequential H_3_PO_4_–CO_2_ assisted synthesis of lignin-derived porous carbon: CO_2_ activation kinetics investigation and textural properties regulation. *Renew. Energy ***191**, 639–648. 10.1016/j.renene.2022.04.036 (2022).

[22] Illingworth, J. M., Rand, B. & Williams, P. T. Understanding the mechanism of two-step, pyrolysis-alkali chemical activation of fibrous biomass for the production of activated carbon fibre matting. *Fuel Proces Technol.* **235**, 107348. 10.1016/j.fuproc.2022.107348 (2022).

[23] Li, Q. et al. Biomass based N-doped porous carbons as efficient CO_2_ adsorbents and high-performance supercapacitor electrodes. *Sep. Purif. Technol.* **275**, 119204. 10.1016/j.seppur.2021.119204 (2021).

[24] Liu, B. et al. Preparation of well-developed mesoporous activated carbon fibers from plant pulp fibers and its adsorption of methylene blue from solution. *Chem. Phys. Lett.* **771**, 138535. 10.1016/j.cplett.2021.138535 (2021).

[25] Dai, Q. et al. Novel granular biomass-based carbons with excellent C_2_H_6_/CH_4_ selectivity for recovering light hydrocarbons from natural gas. *ACS Sustain. Chem. Eng.* **10**, 5633–5642. 10.1021/acssuschemeng.2c00319 (2022).

[26] Hadiya, V. et al. Biochar production with amelioration of microwave-assisted pyrolysis: current scenario, drawbacks and perspectives. *Bioresour .Technol.* **355**, 127303. 10.1016/j.biortech.2022.127303 (2022). 35562022 10.1016/j.biortech.2022.127303

[27] Alfattani, R., Shah, M. A., Siddiqui, M. I. H., Ali, M. A. & Alnaser, I. A. Bio-char characterization produced from walnut shell biomass through slow pyrolysis: sustainable for soil amendment and an alternate bio-fuel. *Energies*. **15**, 1. 10.3390/en15010001 (2022).

[28] Ghodake, G. S. et al. Review on biomass feedstocks, pyrolysis mechanism and physicochemical properties of biochar: state-of-the-art framework to speed up vision of circular bioeconomy. *J. Clean. Prod.* **297**, 126645. 10.1016/j.jclepro.2021.126645 (2021).

[29] Tan, X. F. et al. Sh.-H. role of biochar surface characteristics in the adsorption of aromatic compounds: pore structure and functional groups. *Chin. Chem. Lett.* **32**, 2939–2946. 10.1016/j.cclet.2021.04.059 (2021).

[30] Kwiatkowski, M. & Broniek, E. An analysis of the porous structure of activated carbons obtained from hazelnut shells by various physical and chemical methods of activation. *Coll. Surf. A*. **529**, 443–453. 10.1016/j.colsurfa.2017.06.028 (2017).

[31] Chen, W., He, F., Zhang, S., Xv, H. & Xv, Z. Development of porosity and surface chemistry of textile waste jute-based activated carbon by physical activation. *Environ. Sci. Pollut Res. Int.* **25**, 9840–9848. 10.1007/s11356-018-1335-5 (2018). 29372525 10.1007/s11356-018-1335-5

[32] Yang, P. et al. Porous carbons derived from sustainable biomass via a facile one-step synthesis strategy as efficient CO_2_ adsorbents. *Ind. Eng. Chem. Res.* **59**, 6194–6201. 10.1021/acs.iecr.0c00073 (2020).

[33] Brunauer, S., Emmett, P. H. & &Teller, E. Adsorption of gases in multimolecular layers. *J. Am. Chem. Soc.* **60**, 309–319. 10.1021/ja01269a023 (1938).

[34] Dubinin, M. M. The potential theory of adsorption of gases and vapors for adsorbents with energetically nonuniform surfaces. *Chem. Rev.* **60**, 235–241. 10.1021/cr60204a006 (1960).

[35] Kwiatkowski, M. & Hu, X. Analysis of the effect of conditions of preparation of nitrogen-doped activated carbons derived from lotus leaves by activation with sodium amide on the formation of their porous structure. *Materials*. **14**, 1540. 10.3390/ma14061540 (2021). 33801121 10.3390/ma14061540 PMC8004089

[36] Kwiatkowski, M., Kalderis, D., Tono, W. & Tsubota, T. Numerical analysis of the micropore structure of activated carbons focusing on optimum CO_2_ adsorption. *J. CO2 Util.* **60**, 101996. 10.1016/j.jcou.2022.101996 (2022).

[37] Kwiatkowski, M., Belver, C. & Bedia, J. Effect of synthesis conditions on the porous texture of activated carbons obtained from Tara Rubber by FeCl_3_ activation. *Sci. Rep.* **14**, 2266. 10.1038/s41598-024-52112-5 (2024). 38280927 10.1038/s41598-024-52112-5 PMC10821929

[38] Kwiatkowski, M., He, P. & Valtchev, V. Numerical analysis of the porous structure of spherical activated carbons obtained from ion-exchange resins. *Sci. Rep.* **14**, 102. 10.1038/s41598-023-50682-4 (2024). 38167651 10.1038/s41598-023-50682-4 PMC10761811

[39] Olivier, J. P., Conklin, W. B. & Von Szombathely, M. Determination of pore size distribution from density functional theory: a comparison of nitrogen and argon results. *Stud. Surf. Sci. Catal.* **87**, 81–89. 10.1016/S0167-2991(08)63067-0 (1994).

[40] Lai, W. et al. Artefact peaks of pore size distributions caused by unclosed sorption isotherm and tensile strength effect. *Adsorption*. **26**, 633–644. 10.1007/s10450-020-00228-1 (2020).

[41] Caguiat, J., Kirk, D. & Jia, C. Uncertainties in characterization of nanoporous carbons using density functional theory-based gas physisorption. *Carbon*. **72**, 47–56. 10.1016/j.carbon.2014.01.036 (2014).

[42] Gor, G. Y., Thommes, M., Cychosz, K. A. & Neimark, A. V. Quenched solid density functional theory method for characterization of mesoporous carbons by nitrogen adsorption. *Carbon*. **50**, 1583–1590. 10.1016/j.carbon.2011.11.037 (2012).

