# Author Correction: An analysis of the textural properties of activated carbons obtained from biomass via the LBET, NLDFT and QSDFT methods

**DOI:** 10.1038/s41598-024-80598-6

**Published:** 2024-11-26

**Authors:** Mirosław Kwiatkowski

**Affiliations:** Faculty of Energy and Fuels, AGH University of Krakow, al. Adama Mickiewicza 30, Krakow, 30-059 Poland

Correction to: *Scientific Reports* 10.1038/s41598-024-76297-x, published online 02 November 2024

The original version of this Article contained errors in the Figure legends of Figure 3 and Figure 4. The legends of these figures were inadvertently switched.

The legend of Figure 3:

“The pore size distribution plots obtained for the analysed active carbons via the NLDFT and QSDFT methods.”

now reads:

“The cumulative pore volume plots obtained for the analysed activated carbons via the NLDFT and QSDFT methods.”

The legend of Figure 4:

“Cumulative pore volume plots obtained for the analysed active carbons via the NLDFT and QSDFT methods.”

now reads:

“The pore size distributions plots obtained for the analysed activated carbons via the NLDFT and QSDFT methods.”

The original Article has been corrected.

